# Future of Acute Severe Ulcerative Colitis—A Narrative Review

**DOI:** 10.3390/jcm13247723

**Published:** 2024-12-18

**Authors:** Leshni Pillay, Janakan Selvarajah, Bridgette Andrew, Britt Christensen, Finlay Macrae, Jonathan P. Segal

**Affiliations:** 1 Department of Gastroenterology, Royal Melbourne Hospital, Parkville, Melbourne 3052, Australia; 2 Department of Medicine, The University of Melbourne, Parkville, Melbourne 3010, Australia

**Keywords:** acute severe ulcerative colitis, intravenous corticosteroids, colectomy

## Abstract

While corticosteroids have led to significant reduction in ASUC mortality over the last few decades, they are associated with significant side effects and up to 30% of patients have steroid refractory ASUC, which means we require safer and better therapies for patients with ASUC. Several salvage therapies have been proposed in guidelines; however, we lack high quality head-to-head randomised controlled trials to assess effectiveness and safety of these agents. Furthermore, the role of newer novel agents in ASUC management is unclear. We aim to present an up to date review and envisage future treatment of ASUC without steroids based on current trials and data. In summary, we conclude that ASUC treatment still heavily relies on corticosteroids despite the side effect profile. While infliximab and cyclosporine have extensive data, there are no prospective studies comparing them with corticosteroids as initial therapy. Novel therapies open up the possibility of oral options but require prospective data before any conclusion can be made.

## 1. Introduction

Ulcerative colitis (UC) is a chronic, relapsing–remitting inflammatory bowel disease (IBD) characterised by mucosal inflammation, which usually starts distally and can extend proximally to involve the entire colon [1]. The global prevalence of UC varies widely, with Western regions like North America, Europe, and Oceania having particularly high rates, with estimates indicating around 0.5–1% of the population affected in these regions [2,3]. Overall, however, rates have significantly increased in recent years, especially in previously lower-prevalence regions like Asia [4]. UC has a bimodal distribution with peak incidence between the second to fourth decade of life and a smaller peak in the sixth decade [5].

The exact etiology of UC remains unclear but likely involves the complex interplay between genetic predisposition, environmental triggers, immune system dysregulation, and gut microbiota [6]. Most UC patients experience a mild-to-moderate disease course; however, acute severe ulcerative colitis (ASUC) occurs in 20–25% of patients, representing a serious medical emergency with an associated mortality rate of approximately 1% [7,8,9]. Prior to the introduction of corticosteroid therapy, mortality with ASUC ranged from 22–75% within the first year of diagnosis [10]. Severe complications of ASUC include toxic megacolon and bowel perforation [11], with colectomy rates reaching 40% after multiple severe exacerbations, and with one in five patients requiring colectomy during their initial hospital admission [12].

ASUC was initially described by Truelove and Witts over six decades ago and to date, remains the most widely used criteria for hospitalised patients with UC exacerbation [13]. The modified Truelove and Witts criteria includes the presence of bloody stools occurring six or more times daily in conjunction with any signs of systemic toxicity. These signs include a temperature of 37.8 °C or higher, haemoglobin levels below 10.5 g/dL, an erythrocyte sedimentation rate below 30 mm/h, and/or a pulse rate exceeding 90 bpm [13]. As per the Oxford criteria, if the CRP remains greater than 45 mg/L and/or there is ≥eight bowel movements in 24 h at day 3 of intravenous corticosteroids (ICS), there is an 85% risk of progressing to colectomy [14].

Since the introduction of ICS in the 1950s, medical therapy in IBD has been a field of rapid progression. However, despite the introduction of biologics and other advanced medical therapies in the past few decades, colectomy rates for ASUC have remained steady [9]. In this review, we explore the pre-corticosteroid era, evaluate current ASUC treatment options, and explore the landscape of potential ASUC therapies and propose future strategies to reduce corticosteroid dependency in ASUC management.

## 2. Medical Management of ASUC

Untreated ASUC poses significant risks, including toxic megacolon, bowel perforation, gastro-intestinal haemorrhage, thromboembolism, electrolyte derangement, and risk of colectomy [11]. Currently, the Truelove and Witts ASUC criteria remain the most sensitive clinical tool for predicting clinical outcomes and colectomy risk in ASUC patients undergoing intravenous corticosteroid therapy [13].

A range of clinical indices has been proposed and validated to assess corticosteroid response in ASUC (Table 1). While stool frequency and rectal bleeding are the most consistently used parameters, recent indices have incorporated objective markers such as endoscopic scores and biomarkers, including faecal calprotectin. Table 1 outlines these indices in chronological order to date.

**Figure 1:**
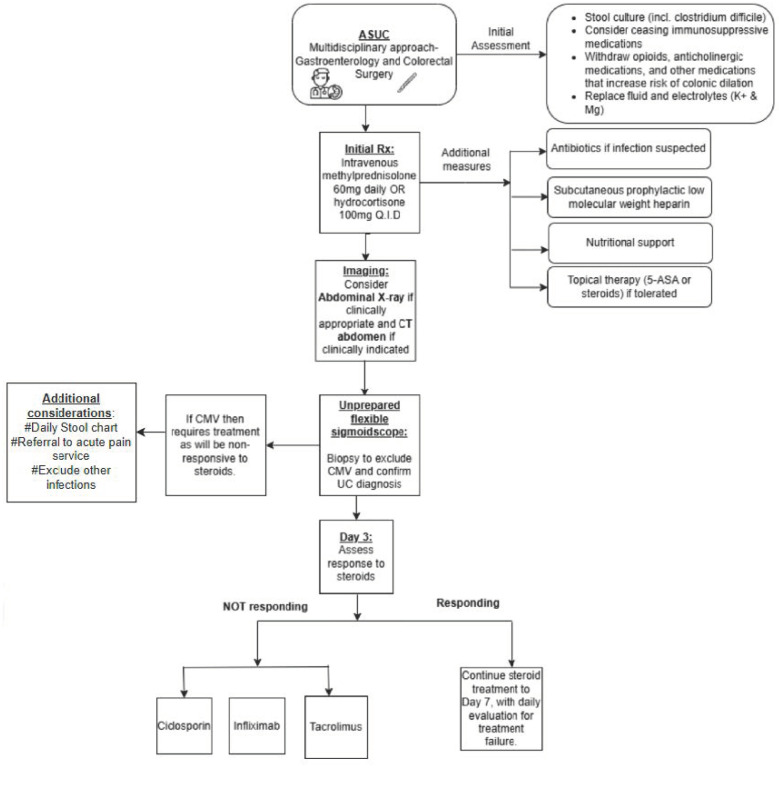
Management pathway for ASUC. Adopted from ECCO Consensus statement 2017 [15]. LEGEND: ASUC—acute severe ulcerative colitis. Rx—treatment. K+—potassium. Mg—magnesium. QID—four times a day. CMV—cytomegalovirus.

The optimal management of ASUC requires a multidisciplinary approach, involving both a gastroenterologist and a colorectal surgeon (Figure 1). Intravenous corticosteroids (ICS) remain the cornerstone of ASUC treatment, following exclusion of secondary causes such as gastrointestinal infections like cytomegalovirus and clostridium difficile (these infections can occur concurrently with underlying UC, therefore complicating the assessment of ASUC). Patients who fail to respond to ICS then proceed to salvage therapies, which include options like infliximab or cyclosporine [15].

## 3. The Pre-Steroid Era

Prior to the introduction of intravenous corticosteroids, mortality with ASUC ranged from 22% to 75% within the first year of diagnosis [9]. The only placebo-controlled trial in ASUC remains the landmark ASUC study in 1955 by Truelove and Witts, which demonstrated that corticosteroids effectively induced clinical remission and reduced mortality, without a significant rise in serious adverse events [13]. In 1974, Truelove and colleagues further established the first intravenous corticosteroid regimen specifically for ASUC [16], and this remains the mainstay of therapy today [17].

## 4. The Steroid Era Continues

After the introduction of ICS regimen in ASUC, short-term mortality rates dropped dramatically to less than 5% [18]. In the modern era, with advancements in IBD therapies, mortality has further declined to less than 1% [19]. Since these first studies by Truelove, there was a paucity of data on long-term outcomes for patients with ASUC treated with corticosteroids, particularly with data being confounded by subsequent salvage therapies.

There are patients with ASUC who respond to ICS and are thiopurine naïve; thiopurines are considered appropriate to maintain clinical remission and the role of infliximab in this setting is not known. An ongoing current randomised controlled trial (RCT)—the ACTIVE trial (EUdraCT 2014-005212-42) aims to answer the question of early infliximab in patients with ASUC who are steroid responsive. In this head-to-head, multi-centre, open-labelled RCT, patients were assigned to combination therapy with infliximab and azathioprine with quick steroid discontinuation or azathioprine and standard steroid tapering regimen. Preliminary results shared at the recent ECCO conference show that combination therapy with infliximab and azathioprine with quick steroid weaning was more effective than combination therapy standard steroid weaning and azathioprine with the primary endpoint being treatment failure at week 52 (defined as any of the following: absence of steroid-free clinical remission, absence of endoscopic response, use of a prohibited treatment, adverse event leading to interruption of treatment, colectomy, or death [20]. Current ECCO guidelines for moderate to severe UC recommend anti-TNF agents for induction in patients who have failed conventional therapies (such as immunomodulators and steroids)). However, despite being a strong recommendation, this is based on moderate-quality evidence [21].

Turner and his colleagues in 2007 demonstrated that colectomy rates over 30 years have remained unchanged even after intravenous corticosteroids were first initiated for ASUC, and after the addition of cyclosporin as salvage therapy for steroid refractory cases [9]. Dosing of steroids in ASUC had been debated, with earlier studies showing no benefit in giving intravenous methyl prednisone doses of greater than 60 mg/day [9,22]. Additionally, intravenous corticosteroid therapy beyond 7–10 days appears to yield no further advantage [23].

While corticosteroids have played a monumental role in reducing ASUC mortality over the last seven decades, they have not proven effective for long-term remission maintenance in UC [24], nor have they significantly reduced overall colectomy rates [9]. With the increasing adoption of STRIDE II guidelines [25] and the treat to target (T2T) approach, it is also essential to note that prior studies show corticosteroids lack significant impact on mucosal healing [26]. Furthermore, significant adverse events can occur with prolonged corticosteroid exposure, which are in Table 2. Even though most steroid adverse events occur after prolonged course of steroid use (i.e., >2–3 weeks), up to 50% of patients can have short-term adverse events [27].

## 5. The Success and Failures of Salvage Therapy in ASUC

At least 30% of those with ASUC do not respond to initial intravenous corticosteroid and require rescue therapy such anti-tumour necrosis factor (anti-TNF), calcineurin inhibitors (CNI), or surgery. Infliximab and calcineurin inhibitors (cyclosporin and tacrolimus) remain the mainstay of salvage therapy in ASUC [34].

Cyclosporine was the first approved salvage therapy following a landmark RCT in 1994 where 11 patients on cyclosporin therapy demonstrated clinical response compared to 9 patients on placebo [35]. Since then, a total of 64 studies involving 2912 patients have been published to date. The studies have been a combination of retrospective, prospective, and RCT trials. Pooled analysis consistently shows that cyclosporin is an effective salvage therapy option with the mean short-term clinical response rate at ~70% (weighted mean; 95% confidence interval [CI] from 68–72%). Response rate was mainly defined as avoidance of colectomy [36]. Despite yielding strong results in the short term, long-term efficacy of cyclosporin in inducing clinical remission is unclear [37].

Cyclosporin has a rapid onset of action, with median time to clinical response of approximately 4 days [35]; if no response is observed by day 7, discontinuation is recommended [36]. Standard cyclosporine dosing is 2 mg/kg, targeting trough levels between 150 and 250 ng/mL [38]. However, cyclosporine’s use is limited due to its significant dose-dependent side effect profile, need for strict therapeutic monitoring, and uncertain long-term benefits. Cyclosporin also has a higher resource burden in patient administration, requiring continuous supervision and protection from sunlight and ultraviolet light [39]. Table 3 outlines reported minor and major adverse events [36].

A second calcineurin inhibitor, tacrolimus, is currently not deemed superior to placebo for steroid refractory UC as highlighted in a recent Cochrane review, published in 2022. This review analysed five RCTs with a total of 347 patients, with only three studies included in the final analysis. The odds ratio for achieving clinical remission and clinical improvement in steroid-refractory UC was 3.76 compared to placebo (95% CI from 1.03 to 14) [40]. Despite previous studies suggesting that tacrolimus was superior to placebo, with colectomy rates comparable to infliximab in steroid refractory moderate-to-severe UC, there remains a lack of data specifically in the ASUC cohort [39]. Furthermore, the role of tacrolimus in maintaining long-term clinical remission as well as colectomy-free survival remains unclear [41].

Infliximab, on the other hand, has been extensively studied as salvage therapy in ASUC. To date, 47 studies involving 2017 patients have been published using infliximab as rescue therapy in steroid refractory ASUC. In a pooled analysis by Gisbert et al., infliximab showed a colectomy avoidance rate of 76% (weighted mean; 95% CI from 75% to 78%). When analysing RCTs alone, the rate was comparable with rate of colectomy avoidance at 67%. Long-term follow-up demonstrated a response rate of 65% with similar results when only analysing the RCTs [37].

The optimal strategy for infliximab, with respect to standard dose (three doses of 5 mg/kg at weeks 0, 2, and 6) versus dose-intensified regimen (induction infliximab at doses up to 10 mg/kg at shorter intervals compared to standard dosing) in steroid refractory ASUC remains a topic of debate. Previous studies show mixed results for dose-intensified infliximab, however three recent meta-analyses did not show that an intensive dosing regimen was superior to standard dosing infliximab with primary endpoints being short- and long-term colectomy rates in ASUC [42,43,44]. These meta-analyses, however, pooled data from retrospective studies with variable infliximab dosing regimens.

The PREDICT UC study by Choy et al., an Australian nationwide, multi-centre, open-labelled, prospective RCT examined standard or dose-intensified regimes (up to three doses of 10mg/kg at standard frequency, with the option of a fourth dose if there was no clinical response) [45]. No significant difference in clinical response or remission was seen at day 14, or at 3 months, though there may be a signal for higher doses in cases with severe hypoalbuminemia [45,46].

Current international guidelines do not support dose-intensified infliximab regimens for ASUC. The international evidence-based consensus on the management of ASUC concluded that the advantage of shorter dosing intervals and/or higher doses of infliximab was unknown [38]. The American Gastroenterology Association (AGA) review in 2020 stated that ‘in hospitalized patients with ASUC being treated with infliximab, the benefit of routine administration of accelerated dosing regimens over standard dosing regimens is uncertain’ [41].

## 6. Cyclosporin Versus Infliximab

Most studies looking at cyclosporin versus infliximab have been retrospective and observational in trial design [37]. There are only two RCTs to date. The cyclosporine with infliximab in steroid-refractory severe attacks of ulcerative colitis (CySIF) RCT is a landmark study that shows treatment failure for standard induction of cyclosporin and infliximab at 60% and 54%, respectively. This difference was not statistically significant [47]. The same group then continued to study the long-term outcomes with a subsequent study over a 5 year period. The primary outcome which was colectomy-free survival at 1 and 5 years for the 2 groups was 71% and 61% for the cyclosporin cohort and 69% and 61% for infliximab-treated patients at 1 and 5 years, respectively (not statistically significant). The authors noted that generally higher proportion of patients required maintenance systemic therapy in the cyclosporin group [48].

The second RCT—comparison of infliximab and cyclosporine in steroid-resistant ulcerative colitis trial (the CONSTRUCT trial)— which was from the UK, was an open-label parallel-group, pragmatic randomised trial, which found no significant differences in the infliximab and the cyclosporine groups for their primary outcome defined as quality-adjusted survival (QAS). Interestingly, the total cost of infliximab was overall higher; regardless of which, there was no statistically significant differences in secondary outcomes of colectomy rates, side effect, and overall mortality. Of note, patients who were randomised to the cyclosporine arm only continued cyclosporin for 6 months. A significant portion of patients were switched to infliximab at the completion of the study [49].

With these two RCTS, it can be deduced that short term outcomes are comparable between infliximab and cyclosporin as salvage therapies; however, to understand long-term outcomes, we need prospective, and double-blinded RCTs.

## 7. Treatment in Infliximab/Cyclosporin-Experienced ASUC Patients

The ideal order and timing of sequential therapy after salvage therapy in ASUC post either infliximab or cyclosporin remains unknown. Patients who fail first-line salvage therapy with either infliximab or cyclosporin, theoretically, can have the other agent as second-line salvage therapy to defer or avoid colectomy. The two major considerations are efficacy of the drug and safety of significant immunosuppression.

Gisbet et al. summarised the previous studies looking at patients with steroid refractory UC who failed either infliximab therapy or cyclosporin/tacrolimus as third salvage therapy and underwent sequential treatment with infliximab therapy or cyclosporin/tacrolimus [36].

### 7.1. Infliximab-Experienced Cohort

Patients who failed infliximab as salvage therapy had sequential therapy with cyclosporin. The studies are summarised in Table 4 [36]. The eight studies examining cyclosporine as third-line salvage therapy show mean colectomy-free rates with infliximab followed by cyclosporine of 42%.

### 7.2. Cyclosporin/Tacrolimus-Experienced Group

Patients who failed cyclosporin/tacrolimus as salvage therapy had sequential therapy with infliximab. The 14 studies examining infliximab as third-line salvage therapy show that the mean colectomy-free rate with cyclosporin followed by infliximab was 58%, which was not an appreciable difference to the alternate sequence. This is summarised in Table 5 [36].

Now, changing the focus to safety of sequential immunosuppression in ASUC: from the studies summarised in Table 4 and Table 5, the overall mortality rate was 3 (grey highlight Table 5) out of total 201 patients in the 14 studies from the group having cyclosporin/tacrolimus first, followed by infliximab. Causes for death were nosocomial pneumonia (Chaparro et al.) [50], pulmonary embolism (Leblanc et al.) [51], and sepsis (Maser et al.) [52]. Studies examining patients who underwent salvage therapy with infliximab first and then sequential therapy with cyclosporin did not report any deaths. These studies, despite being very insightful, however, in the setting of previous trial design (mainly retrospective with small numbers, unblinded, and lack of RCTs) as well as no current studies having comparable data with risks of colectomy versus risks of sequential immunosuppression, make it challenging to deduce the role sequential immunosuppression vs. proceeding to colectomy after first-line salvage therapy.

Given these challenges, many researchers are exploring alternatives for salvage therapies. The interleukin 1 (IL-1) blockade in acute severe colitis (IASO) trial team recently published their results investigating the role of anakinra as a rescue therapy in ASUC [53]. They performed a phase II, multi-centre, randomised (1:1), placebo-controlled, double-blinded trial of anakinra, given concurrently with IV corticosteroids in patients hospitalised with ASUC. Logistical modelling showed that the need for rescue therapy would not be reduced with anakinra treatment; the trial was terminated early as it was a negative study [53]. Despite previous studies showing that IL-1 is perhaps a key mediator in colonic inflammation with its mechanism involving local activation of neutrophils and downstream inflammatory mediators [54], the IASO group have demonstrated that IL-1 is likely not a therapeutic target in ASUC.

## 8. Proposed Newer Therapies

### 8.1. Ustekinumab

In three previous retrospective studies, ustekinumab was used in combination with either cyclosporin or tacrolimus in patients previously exposed to a biologic agent (anti TNF, vedolizumab) where all patients were able to achieve colectomy-free remission in the short term. The first study reported a patient with steroid-refractory, biologic, and JAK –2 nonresponsive ASUC who had cyclosporine re-initiation combined with ustekinumab. He completed a total of 3 months of cyclosporin therapy. Following this, he was continued on subcutaneous ustekinumab and deemed to be in clinical remission at 6 months [55]. Similarly, the other two studies demonstrated that ustekinumab can be considered as an adjunct therapy in combination with calcineurin inhibitors to potentially avoid colectomy [56]. An added advantage of ustekinumab is its relatively good safety profile [57].

### 8.2. Tofacitinib

Since the introduction of smaller molecules in IBD, there has been much interest in the role of these newer agents as potential steroid-sparing agents in ASUC and for consideration as third-line rescue therapies to reduce overall colectomy rates. The features of JAK inhibitors that demonstrate the potential are many, as listed in Table 6.

To date, 19 studies have been published assessing the efficacy of tofacitinib in ASUC. Most of these studies were retrospective and observational. The only RCT published to date is the tofacitinib in acute severe ulcerative colitis study (TACOS study), which was a single-centre, double-blinded, placebo-controlled RCT, where a total of 104 patients on intravenous corticosteroid (100 mg of hydrocortisone) were randomised to placebo and tofacitinib arms. At day 7, clinical response was achieved in 83.01% patients in the tofacitinib arm compared to 58.82% in the placebo group (odds ratio of 0.27, CI 0.09–0.78, *p* value of 0.01.) The study overall showed a good safety profile with only one patient reported to have dural sinus thrombosis [62].

Eqbal et al. performed the longest follow-up for steroid-refractory ASUC patients treated with tofacitinib post failing infliximab. In their study, they used high-dose tofacitinib at 10 mg TDS. A fairly high rate of clinical and biochemical response was reported in 10 out 11 patients, with only 2 patients progressing to colectomy. The safety profile of tofacitinib was comparable to previous studies and there were no major adverse events reported [63].

### 8.3. Upadacitinib

Berinstein and colleagues in a multi-centre prospective cohort study with 25 patients receiving upadacitinib in combination with intravenous corticosteroids for ASUC showed a colectomy-free rate of 76% at 90 days. Secondary outcomes showed an impressive steroid-free remission rate of 83% [64]. The same group more recently reported the role of upadacitinib as a second-line salvage therapy in biologic naive patients failing intravenous corticosteroids and third-line salvage therapy in patients who failed intravenous corticosteroids and previous biologics. The study was conducted as a prospective, cohort study in four patients and the overall clinical outcomes were similar to upadacitinib being used as a second- or third-line salvage agent [65]. To date, there are 11 published studies on the role of upadacitinib as a salvage agent in ASUC [66]. However, we lack any prospective RCTs to confidently predict the short- and long-term efficacy of upadacitinib.

### 8.4. Vedolizumab

Vedolizumab is a selective, humanised immunoglobulin G1 monoclonal antibody to α4β7 integrin, mediating its mechanism of action by blocking lymphocyte trafficking to gut mucosa [67]. Vedolizumab is effective in induction of clinical response and maintenance of clinical response in moderate-to-severe UC as per the landmark GEMINI trial [68]. Similar to ustekinumab, there are no current RCTs to date exploring the role of Vedolizumab in ASUC. Previous studies summarised by Gisbert et al. show that most studies (six out of eight) were small and retrospective, and in almost all of the studies, vedolizumab was used as combination therapy with cyclosporin or tacrolimus [36]. To date, the largest study for ASUC patients treated with vedolizumab by the Chicago group shows that vedolizumab was effective as being used as maintenance therapy after initial treatment with combined calcinuerin inhibitor and vedolizumab. The colectomy-free survival rates in this group of 71 patients was 93% at 3 months, 67% at 1 year, and 55% at 2 years [69].

## 9. Surgical Approach

Despite the use of salvage therapy like calcineurin inhibitors or infliximab, colectomy rates remain close to 30% for patients presenting with ASUC [10]. Generally, in UC, there are three main indications for surgery: acute severe colitis, refractory UC, and associated dysplasia and carcinoma [70].

The most commonly performed initial operation in ASUC is a semi-elective and staged laparoscopic subtotal colectomy with Brooke ileostomy, with preservation of the rectal stump [71]. Subtotal colectomy with Brooke ileostomy has many advantages—it being a purely abdominal procedure, therefore eliminating the need for pelvic dissection. Additionally, it allows comprehensive pathological evaluation of the colonic specimen in uncertain cases. There are some drawbacks to this procedure—the main one being the risk of long-lasting or permanent stoma formation. Managing the rectal stump remains a critical consideration; creating a mucous fistula by opening the rectal stump’s tip may mitigate pelvic sepsis risk and reduce postoperative morbidity. This can be positioned in the left lower quadrant, at the median laparotomy incision’s end, or through the same aperture as the ileostomy, leaving a prolonged rectal/rectosigmoid stump. The latter requires patients to manage an additional stoma until definitive surgical intervention [70].

Post-subtotal colectomy, there remains an option (in selected patients) to proceed to completion proctectomy with ileal pouch–anal anastomosis (IPAA) within 6–12 months [71,72]. Restorative proctocolectomy with IPAA was first introduced by Parks and Nicholls in 1978 and still remains the preferred surgical approach for elective treatment of ulcerative colitis in patients with intact sphincter function and no identifiable risk factors for post-operative complications [73]. The main advantage of IPAA is avoidance of a permanent stoma, acceptable functional results, and overall good quality of life. Major considerations for IPAA is the future likelihood of stricturing disease and the chance of sexual impairment and infertility [70].

Total colectomy with ileorectal anastomosis (IRA) remains a selective alternative to ileoanal pouch (IPAA). It may be considered in young women in their reproductive years (due to its avoidance of extensive pelvic dissection, thereby reducing the risk of sexual and urinary dysfunction) [74]. Indications for IRA are restricted to patients with minimal rectal disease activity and relative contra indications are patients with poor sphincter function, severe rectal disease, or noncompliant rectums [75]. Given the risks of rectal cancer and persistent inflammation, lifelong surveillance of the retained rectum is essential.

When performed in a semi-elective setting, colectomy is a safe, feasible, and definitive option for those who have elected it for lifestyle measures with a permanent stoma, or exhausted medical options [72]. Prolonged steroid treatment can delay necessary surgical intervention, increasing perioperative morbidity and mortality, with infections rather than bowel perforation becoming the primary cause of death [10]. When colectomy is performed beyond day 7 of admission, or in an urgent, as opposed to semi-elective, setting, there is a significantly higher risk of perioperative complication and mortality [1,10,72]. Early surgical assessment, standardised treatment algorithms, and predictive scores can reduce the need for emergency surgery and improve patient outcomes [1,72].

Over recent years, minimally invasive surgery (MIS) has become an acceptable alternate to open surgery in UC. Other newer techniques, such as robotic surgery and single-incision laparoscopic surgery (SILS), have already been shown to demonstrate success in ileal pouch–anal anastomosis (IPAA) procedures. However, robotic surgery carries higher costs compared with conventional multiport laparoscopic approaches, while SILS requires advanced expertise, making it more suitable for experienced laparoscopic surgeons [71]. While MIS represents the future of colorectal surgery, further studies are necessary to define the optimal role of these techniques within treatment algorithms, particularly in ASUC.

In ASUC, while toxic megacolon commonly necessitates consideration for surgical intervention, some patients may not fulfill clinical or imaging criteria for megacolon but instead may show signs of ‘impending’ megacolon [76]. “Impending megacolon” precedes development of toxic megacolon and is thought to be due to gaseous dilation of the uninflamed small bowel from a distally inflamed large bowel [77]. It is characterised by abdominal X-ray finding of persistent small bowel distension [76]. Despite impending megacolon not being well established in the literature, it may be a predictor of toxic megacolon [76] and, therefore, may provide a useful window of opportunity for therapeutic intervention.

## 10. Future of ASUC Treatment Options

Acute severe ulcerative colitis (ASUC) remains a formidable clinical challenge, characterised by rapid progression, high morbidity, and frequent need for hospitalisation and intensive therapy. While current management strategies—comprising corticosteroids, biologics, and surgical interventions—have improved outcomes, gaps in efficacy and treatment response underscore the urgent need for novel therapeutic approaches. The future treatment landscape is likely to focus on precision-targeted therapies, advanced biologics, and personalised medicine strategies to address the diverse clinical presentations and variable responses observed in ASUC.

Targeted Immunotherapy: Current standard therapies for ASUC predominantly involve systemic corticosteroids and biologics targeting pathways such as tumour necrosis factor-alpha (TNF-α), integrins, and interleukins. However, emerging evidence suggests that refined immunotherapy targeting specific immune pathways could enhance treatment outcomes. Janus kinase (JAK) inhibitors, including upadacitinib and tofacitinib, are being investigated for their ability to modulate intracellular signalling pathways implicated in inflammation. Early data from other ulcerative colitis subsets indicate that these agents may represent a promising option for patients with corticosteroid-refractory ASUC.

Novel Biologics and Cellular Therapies: Next-generation biologics targeting specific immune pathways represent a growing area of promise. Agents directed against interleukin-23 (IL-23), interleukin-17 (IL-17), and other cytokines involved in the inflammatory cascade are currently under evaluation. Biologics such as ustekinumab (anti-IL-12/23) have demonstrated efficacy in other forms of ulcerative colitis and may serve as frontline therapies for ASUC, particularly for those unresponsive to TNF inhibitors.

Personalised Medicine and Biomarker-Guided Therapy: Precision medicine offers the opportunity to tailor treatment to individual patient profiles by leveraging biomarkers, genetics, and epigenetic signatures. More recently, the role of the urotensin-II receptor (UTR) in acute severe ulcerative colitis (ASUC) and steroid responsiveness is an emerging area of interest. Urotensin-II is a peptide with potent pro-inflammatory effects, acting through the UTR to mediate immune responses, smooth muscle contraction, and inflammation and recent evidence suggests that elevated UTR expression may contribute to the pathophysiology of ASUC by promoting cytokine release, immune cell recruitment, and intestinal inflammation [78]. Furthermore, UTR modulation may influence steroid responsiveness; altered UTR signalling could impair the immunosuppressive effects of corticosteroids, contributing to treatment resistance in some ASUC patients [79]. Understanding the UTR pathway’s role may help predict steroid responsiveness and identify new strategies to enhance therapeutic outcomes. Such newer approaches may promise earlier identification of high-risk individuals and targeted use of biologics and immunosuppressants, improving efficacy while minimising side effects and unnecessary treatment exposure.

Advances in Surgical Interventions and Minimally Invasive Techniques: For patients with refractory or life-threatening ASUC, surgical intervention remains critical. The future will likely see advances in minimally invasive surgical techniques and robotic-assisted approaches, which aim to reduce complications, shorten recovery times, and improve surgical outcomes. Innovative surgical strategies focused on sphincter preservation and reducing long-term complications could further enhance quality of life for patients with severe, treatment-refractory disease.

*Combination Therapies*: Combining therapeutic strategies—such as biologics with immunosuppressants or interventions targeting both immune and microbial pathways—may offer improved efficacy and reduce the risk of treatment resistance. Combination approaches could simultaneously address multiple pathogenic mechanisms, enhancing the likelihood of clinical remission and sustained disease control.

## 11. Conclusions

Our review demonstrates that corticosteroids remain the mainstay therapy for ASUC despite reported adverse events with their use. Infliximab and cyclosporine are well studied for salvage therapy with head-to-head comparison trials but their use is limited by lack of clarity on dosing, need for drug level monitoring, and lack of large scale trials comparing them with corticosteroid therapy upfront. Novel therapies such as tofacitinib provide many advantages such as rapid onset of action and elimination and prompt clinical responses, as well as being cost effective. Despite our advances, colectomy rates are still high in ASUC and medical therapy should be rapidly switched to surgical management if not responding, which requires active daily collaboration with surgical colleagues. We propose that the future treatment paradigm for ASUC will be to prioritise targeted immunotherapy, personalised approaches guided by biomarkers, and innovative surgical strategies. Continued research into these evolving modalities is essential to overcome the challenges of ASUC, improve patient outcomes, and address unmet clinical needs in this life-threatening condition.

## Figures and Tables

**Table 1:** Clinical indices for predicting IV corticosteroids refractoriness in ASUC [14].

Predictive Index (for Adults)	Parameters	Cut-Off for Steroid Refractoriness on Day 3 Unless Otherwise Stated
Oxford (1996)	Bowel movements, CRP *	>8 bowel movements or 3–8 bowel movements with CRP * > 45.
Lindgren (1998)	Bowel movements, CRP *	[CRP * (mg/L) × 0.14 + bowel movements] > 8.
Seo (2002)	Bowel movements, ESR **, Hb ^, Albumin	[60 × number of bloody stool + 13 × bowel movements + 0.5 × ESR ** (mm/h) − 4 × haemoglobin (g/dL) − 15 × albumin (g/dL) + 200] > 200 on day 14 (predictive for inpatient colectomy).
Ho (2004)	Bowel movements, colonic dilatation, serum albumin	Score 0–9 for each variable: bowel movement, colonic dilation (>5.5 cm), hypoalbuminaemia. Scores of 0–1, 2–3, and ≥4 had a medical therapy failure rate of 11%, 43%, and 85%, respectively.
Edinburgh score (2004)	Bowel movements, colonic dilatation, serum albumin	Mean bowel movements on day 3 (0–4 points), hypoalbuminaemia (<30 g/L: 1 point), colonic dilation (>5.5cm: 4 points). Composite score ≥ 4.
AIMS (2017)	UCEIS ^#^: Vascular pattern (0–2) Bleeding (0–3) Erosions and ulcers (0–3)	UCEIS ^#^ > 6 on admission FCP ^$^ > 1000 µg/g.
CRP/Albumin ratio (2018)	CRP *, serum albumin	CRP */Albumin > 0.85.
ACE Score (2020)	CRP *, serum albumin, endoscopic assessment (Mayo score)	CRP * ≥ 50 mg/L (1 point), serum albumin ≤ 30 g/L (1 point), endoscopic—Mayo score = 3 (1 point). Composite score > 3.
ASUC score (2020)	Serum albumin, steroid use, UCEIS ^#^ (vascular pattern, bleeding, erosions/ulcers)	At least 2 of: S. albumin ≤ 30 g/L, steroid use, and UCEIS ^#^ > 6 on admission.
ADMIT-ASC score (2022)	CRP *, serum albumin, UCEIS ^#^ (vascular pattern, bleeding, erosions/ulcers)	CRP * ≥ 100 mg/L (1 point), serum albumin ≤ 25 g/L (1 point), UCEIS ^#^ ≥4 (1 point) or ≥ 7 (2 points). Composite score ≥ 3 on admission.

* CRP = C-reactive protein ** ESR = erythrocyte sediment rate ^ Hb—haemoglobin ^#^ UCEIS = ulcerative colitis endoscopic index of severity ^$^ FCP = faecal calprotectin.

**Table 2:** Adverse events secondary to steroids in UC.

	Adverse Event	References
Secondary to supra physiological dosing	Infection (sepsis), anaemia, cellulitis, varicella, herpes zoster and scabies, increased risk for Hepatitis B reactivation.	[28,29]
	Hyperglycaemia.	[30]
	Dermatological: acne, skin bruising, skin thinning, striae, poor wound healing, moon face, oedema.	[31]
	Psychiatric: mood changes, insomnia, psychosis.	[32]
Prolonged use	Infection.	[28,29]
	Osteoporosis 17–41%.	[33]
	Avascular femoral osteonecrosis.	[33]
	Myopathy.	[33]
	Cataracts, glaucoma.	[33]
	Cushingoid features, hypertension, hypokalaemia, weight gain, fatty liver	[26,33]
	Adrenal insufficiency.	[31]
Withdrawal complications	Corticosteroid withdrawal syndrome.	[31]

**Table 3:** Adverse events (AE) with cyclosporin.

Minor AE (30–50%)	Major AE (up to 17% of Cases)
Hypokalaemia	Hypertension
Hypocalcaemia	Nephrotoxicity
Tremors	Opportunistic infections
Paresthesias	Neurotoxicity
Malaise	
Headache	
Liver function tests derangement	
Gingival hyperplasia	
Hirsutism	

AE—adverse events.

**Table 4:** Cyclosporine as a third-line rescue therapy after infliximab failure (reproduced from Gisbet et al. [36].

Author	Sequence of Treatment	# of Patients	Median Follow-Up (Months)	Colectomy-Free Rate (%)	Adverse Event (%)
Chapman	Infliximab–cyclosporine	1	9	100	0 [0 deaths]
Gornet	Infliximab–cyclosporine	4	10	25	30 [0 deaths]
Leblanc	Infliximab–cyclosporine	21	22.6	34	24 [0 deaths]
Maser	Infliximab–cyclosporine	9	28.5	60	22 [0 deaths]
Mocciaro	Infliximab–cyclosporine	2	33.6	0	0 [0 deaths]
Naves	Infliximab–cyclosporine	2	44	50	0 [0 deaths]
Protic	Infliximab–cyclosporine	2	12	0	NA * [0 deaths]
Weisshof	Infliximab–cyclosporine	40	13	42	40 [0 deaths]

* NA—not applicable.

**Table 5:** Infliximab as a third-line rescue therapy after failed clinical response to cyclosporin/tacrolimus (reproduced from Gisbet et al. [36]).

Author	Sequence of Treatment	# of Patients	Median Follow-Up (Months)	Colectomy-Free Rate (%)	Adverse Event (%)
Bernardo	Cyclosporine–infliximab	1	NA	100	NA
Chaparro	Cyclosporine–infliximab	47	12	70	23 [1 death]
Gornet	Cyclosporine–infliximab	5	10	60	30 [0 deaths]
Herrlinger	Tacrolimus–infliximab	24 [19 ASUC]	10	42	33 [0 deaths]
Lam	Cyclosporine–infliximab	1	4	0	0 [0 deaths]
Leblanc	Cyclosporine–infliximab	65	22.6	46	23 [1 death]
Mañosa	Cyclosporine–infliximab	16	47	63	19 [0 deaths]
Maser	Cyclosporine–infliximab	10	28.5	56	10 [1 death]
Mocciaro	Cyclosporine–infliximab	1	NA	100	0 [0 deaths]
Naves	Cyclosporine–infliximab	6	44	83	17 [0 deaths]
Protic	Cyclosporine–infliximab	10	12	90	NA * [0 deaths]
Protic	Tacrolimus–infliximab	2	12	100	NA * [0 deaths]
Tsukamoto	Tacrolimus–infliximab	1	30	0	NA * [0 deaths]
Yamamoto	Tacrolimus–infliximab	12	16	58	17 [0 deaths]

KEY: * NA—not available.

**Table 6:** Properties of tofacitinib.

Properties of Tofacitinib	Evidence
Pharmacokinetics	Oral administration with a bioavailability of 74% [58]. Rapid absorption with peak concentrations reached in 30–60 min and steady state in 24-48 hrs [59]. Rapid elimination due to hepatic and renal excretion with a half-life of 3 h and up to 95% of drug elimination by 24 h [59] vs. half-life of infliximab—9–12 days [59]. Likely reduced risk of infection and poor wound healing.
Clinical response	Clinical response by day 3 in a phase three post hoc study [60].
Immunogenicity	Not known in JAK 2, therefore there is no need for additional immunomodulator therapy, unlike with anti TNF.
Patient factors	Patients generally prefer oral therapy but no specific trials have addressed IV vs. oral.
Affordability	Tofacitinib costs per year is cheaper than infliximab–remicade but more expensive than inflectra (infliximab) [61]. Must be noted that affordability varies depending on location and commercial market forces.

## Data Availability

No new data were created or analysed in this study. Data sharing is not applicable to this article.
